# Ninth Annual Meeting of the American Society of Dental Surgeons, Held at Saratoga, August, 1848

**Published:** 1851-01

**Authors:** William Henry Burr

**Affiliations:** Phonographer


### NINTH ANNUAL MEETING OF THE AMERICAN SOCIETY OF DENTAL SURGEONS, HELD AT SARATOGA, AUGUST, 1848.

REPORTED BY WILLIAM HENRY BURR, PHONOGRAPHER.

*Continued from October No.*

I next heard that Dr. Brewster had used India rubber for separating the teeth. My early experiments with this substance produced nearly as much inflammation as my experiments with wood. I continued, however, the experiments, and in two cases succeeded in separating the teeth with scarcely any inflammation. This encouraged me to pursue the experiments, which I did, with the following results: I found that by carefully graduating the sizes of the pieces of rubber used, the teeth might be separated a considerable distance without producing much inflammation. I cut the Indian rubber to the requisite thickness at the time it is to be applied, which can easily be done with scissors. For some time past, I have prepared the rubber for use, by boiling it in water until it becomes quite soft. When thus softened, it is superior to any substance, with which I am acquainted for this purpose. When the rubber is thus made soft, the force is applied so gently, that there is very little, if any inflammation to be apprehended from its use. It takes, I am free to confess, longer time to separate the teeth by means of boiled rubber, than either by wood or by the rubber unboiled. But as it is of the utmost importance to prevent inflammation of the periosteum during this operation, I less regret the delay. The rubber after boiling, is quite as elastic as it was before; but its mechanical force is considerable diminished. If I separate the teeth for the purpose of filling on the approximating surfaces, and wish them afterwards to resume their original position, I reduce the thickness of the rubber, day by day until they shall have returned to their original place. It is quite as desirable that the teeth should come together gradually, as it was desirable that they should be separated gradually. This has been my practice, and the results have fully answered my expectation. I should be happy, however, to be instructed by the suggestions of others upon this subject, as to me, it has ever been an exceedingly interesting one.

Dr. Foster inquired of Dr. Rich, if he could always manage to get the soft rubber between the teeth ?

Dr. R. replied, that the soft rubber would be introduced between the teeth much more easily than the hard. When it was found difficult to introduce the rubber, on account of the teeth being close together, it was his (Dr. R's) practice to pass floss silk waxed between them and to allow it to remain between a few minutes. After which the rubber might be introduced without difficulty. As regards the operation of filing it was Dr. R's practice to file or cut away the surfaces until the edges of the cavities were firm enough to bear the pressure of filling. He would not leave a cavity to grow larger before it was filled, nor would he hesitate to file away the surface on account of a cavity being small. If decay exists in any part of a tooth, and that decay cannot be cut or filed away without disfiguring the tooth, it ought to be filled at once. The smaller the cavity, the greater would be the service rendered to the tooth by filling. If the decay be left in order that the cavity may become larger, the caries may extend in the direction of the cavity of the nerve, instead of spreading over a superficial surface.

Dr. Westcott said that he had not yet met with a case where he could not determine whether the operation of filing should be performed at once, or left for the cavity to increase in size. If there was any doubt about it, it was better to operate early than to leave it till the nerve was exposed. With regard to the material used for separating the teeth, Dr. W. had seen an instance in which white wax had been used by a lady to supply the place of a lost tooth, and it had, by continual renewal, gradually increased the space between the teeth so as to leave ample room for another tooth: she had worn the wax for eight years, and the gum was perfectly healthy.

Dr. Dwinelle thought Dr. Rich was rather too particular and nice in the matter of separating teeth, and of reducing them back again. The necessity of gradually reducing them back, was not very apparent to him, because they would naturally return to their places gradually.

Dr. Dunning said that he had used India rubber but little, having found it exceeding difficult to make it retain its place between the points of teeth that approximated very closely. In such cases he was in the habit of using a very small quantity of cotton, for the purpose of commencing the separation, which was completed by wedges of wood. The cotton is introduced by the aid of a thin and very smooth instrument of steel, which forces it to the desired position between the teeth.

Dr. J. Parmly had not found any particular difference in the materials used, except when greater pressure was produced. He had performed a great many operations of separating the teeth, and had never found them to result in the death of the teeth. By a certain pressure with a steel instrument, you may destroy the tooth, and it will not show till perhaps a month after, but I have never seen a tooth destroyed by wedges. In moving the tooth, its connection with the nerve cannot be destroyed. In no case can the teeth be moved, as far as my experience goes, without some pain. If the amount of pressure is produced which will separate the teeth, some pain must be produced. I have known several cases where the teeth have separated without any pressure. In one case where I extracted a tooth, I found two-thirds of the whole membrane destroyed, and the outside adhering closely upon drawing the tooth out.

Dr. Foster cited a case of a young lady of thirteen years of age, whose upper front incisors projected very far out beyond the under.

The space between these teeth had become very wide, and after regulating them and confining them in their proper position, he found the antagonizing teeth did not touch them upon their inner surfaces, nor was there any evidence that they had done so. The gradual departure in this case from the natural position, had been caused by a constant habit which she indulged in of keeping her thumb in her mouth, and exerting contionual pressure upon these teeth outward.

Dr. Rich. Few will deny that it would be mal-practice to force the teeth suddenly into the position desired. In my opinion it is the wiser practice to move them in the slowest manner possible, nor do I imagine that I shall be found singular in this opinion.

Many practitioners entertain a notion that the teeth cannot be separated without pain. Experience has taught me the reverse, provided always the separation be effected gradually and gently. If during this process of separating, where I see the teeth every day, and am informed by the patient that there is no pain, and if upon examining the parts, I detect no inflammation, it is to my mind the best possible evidence that my theory and practice are correct; more especially, when this state of things occurs again and again in different individuals. One incontrovertible fact outweighs all the improved theories that can be advanced.

I have often moved teeth considerable distances in young persons, when remedying irregularities without pain to the patient. In one operation of this kind, which was performed without pain, I was obliged to move a central incisor nearly a quarter cf a circle before it was brought within the line of the arch. The patient was a lad 15 years old, and when I first saw the case, the tooth projected out of the mouth; so that the lip was raised by it considerably. Still the operation was performed with so small an amount of inflammation or inconvenience, that after the operation was completed, the boy said to his father—"I do not think it required much skill to fix my teeth; I could have done it myself if I had had a piece of India rubber and some gold wire." I can conceive no greater compliment to a practitioner, than was conveyed in this assertion, that the skill of the dentist could render a most difficult and generally painful operation so apparently easy that the child should have thought that he could have done it himself.

Good evidence that the teeth can be moved from their original position without pain, may also be found in the fact, that the teeth, particularly the incisors, often change their position of themselves moving to the right or left and leaving large spaces between the teeth, or projecting out in front. If we question the individuals with whom this has occured, we shall be told that the change has taken place gradually and to themselves imperceptibly. Repeated observations have convinced me these alterations in the position of the teeth are brought about by pressure of the antagonizing teeth and occur where the whole force of the jaw is brought to bear upon one tooth either by the loss or wearing down of the back teeth or by the swelling of the periosteum of the displaced tooth. In order to restore teeth which have been thus displaced to their proper position, I have always found it necessary to commence by filing the antagonizing surfaces so that they could no longer come into contact.

Dr. Foster thought with Dr. Rich, that the softer the substance used, the less would be the irritation. He had some cases in remembrance of patients who always separated their own teeth, and preferred doing so, succeeding quite as well in making wide spaces in less time, and as they said, with less pain, by compressing a pledget of cotton between the teeth.

Dr. Nelson. It is astonishing how slight a pressure will separate the teeth. With regard to the teeth returning again to their place, Dr. Rich's practice differs from mine, which has uniformly been to let the teeth return again as soon as possible. If I separate them, I immediately apply a force to the lateral sides. If you draw a piece of rubber between your teeth, it necessarily produces pain; if you remove it, the pain subsides. With regard to filing, I am satisfied that in many instances it is the only mode of saving the teeth. It is a bad practice, however to leave the filed surfaces touching each other. I know a lady whose teeth were filed quite half away twenty-five years ago, and there is no sign of decay. The practice of separating by wood does not commend itself to me, because the wood seems to expand rapidly from the moisture, and produces a great deal of pain. Rubber causes less pain; cotton acts slowly, and has a happy effect. If the cavities are large, extending under the gums, the points of the latter are pressed away for the time being, and gives the operator an opportunity to fill.

Dr. Dunning thought cotton was only preferable when the surfaces approximate so closely and the edges were so sharp that wedges would be likely to break in introducing them.

Dr. Rich described his method of preparing India rubber for separating the teeth. I use the natural or white rubber, and prepare it for use by boiling it eight or ten hours. The rubber requires to be kept moist and warm after boiling, or it will become hard again in a few hours. Moreover, this process changes the color of the rubber, making it nearly white, so that it does not disfigure the mouth by presenting a dark mass between the teeth.

Dr. Westcott wished to be understood as to what he had said with regard to leaving cavities to increase, so as not to be obliged to file away too much. No matter how small the cavity was, if it was deep, it ought to be filled immediately. Not unfrequently the cavity was so slight as not to warrant the disfiguring of the teeth by filling. He had in his mind such cavities on the convex surfaces of molar teeth. A week since he was called on to fill such a cavity, between the wisdom tooth and second molar. Upon examining the cavity he told the patient to let it go for the present, and by and by it could be filled without as much filing as now. In regard to small cavities in the front teeth, Dr. W. uniformly separated by wedges, prefering to have one uniform surface.

Dr. Dunning considered the subject of practical dentistry of thrilling interest, and expressed his gratification for the rich treat he had enjoyed, in common with the rest, in the interchange of thoughts on this important subject.

Dr. Harris. As the operation of separating teeth with wedges of wood, or some other sufficiently hard substance, originated with the President of this Society, if I am correctly informed, it would give me pleasure, as I doubt not, it would all the other members of the Association, to hear his views upon the subject.

The afternoon being exhausted, the further discussion was adjourned till the next day.

SECOND DAY, 3½ P. M.

The chairman being requested by Dr. Harris to give some information with regard to the origin of the use of wedges, for separating the teeth, spoke as follows:

Permit me to say, that I have never attended any meeting of the society in which I felt an equal degree of pleasure and interest to that I did yesterday in the discussion of subjects connected with practical dentistry. If we can carry on the zeal and good feeling with which we have commenced, we shall attain to the high objects which first led a few individuals to the establishment of this association, improve our knowledge and advance the interests of others, at the same time, with our own.

It was remarked by Dr. Harris, that he believed I was the first who introduced the practice of using wedges. In the year 1820 I lost the crown of the first bicuspid tooth, and I had one engrafted upon its root, which I wore about four years, when the root becoming troublesome, I had it extracted. I then had one adapted to a gold plate, which I wore for the same length of time, when the adjoining root round which the spring was secured became tender from the contact of the spring, or from other causes. It then occurred to me that the second bicuspid might by pressure be made to take the place of the first, and in 1832, being in the country and having time to attend to it, I commenced the operation by introducing a small wooden wedge between that tooth and the first molar, and by inserting a larger wedge each subsequent day, I found at the end of about four weeks that the crown had completely taken the place of the one previously lost. By wearing the wedge for a length of time, from which I suffered no pain or inconvenience, the tooth maintained its position, and so continued for several years. On my describing the sensitiveness in the neck of the tooth, occasioned by the abrasion of the spring, to Dr. S. Brown, he proposed covering or protecting the exposed surface with mineral paste, which he said he had discovered a new mode of preparing, and a few days afterwards the paste was applied by Dr. B. In a short time the tooth became exceedingly painful, and I had it extracted. This was my first and only experience with mineral paste. The result of its application in my case being almost immediate inflammation of the gum and membrane, from which I lost the tooth.

On my return from the country in 1832, I commenced operating by means of wedges in front teeth, instead of filing them as I had previously done, and I continued to do so, believing it to be the most prudent and successful method of separating front teeth for the purpose of stopping on their contiguous edges or sides.

After I had been in this practice about a year or so, an incident occurred which led me to the belief that such a mode of procedure was not known and had never been performed until I adopted it. Miss T., then about twelve years old, came to me; her front teeth were decayed, there being cavities in depth about twice the thickness of the enamel. Her mother asked me if I thought it best to file, in order to stop them. I replied, that in so young a person, I did not deem filing advisable, and that I would open them sufficiently by means of wedges and then stop them, and that when stopped, the teeth would immediately fall together again. The mother said that she had heard of my op erating in that way for a young friend of her daughter's; and that she had called upon her dentist that morning to have her child's tooth filled by him in that way, when he asserted, "Madam, it cannot be done."

The lady replied, "Mr. Parmly has done it for a young friend of her's." The dentist, with great and emphatic earnestness, reiterated—"Mr. Parmly may say he has done it, but it cannot be done." After such a marked display of professional courtesy, I felt exceedingly interested in the matter, and with the lady's consent, I began wedging, and in a week or so, completed the operation by carefully stopping the two teeth. I then requested her to show them to her dentist, to prove to him that it could be done.

From that day to this, I have uniformly followed this practice in all cases where it would have been necessary after filing to cut into the sound portion of the tooth for the purpose of getting sufficient depth to retain the stopping, or where the nerve by such cutting might be laid bare, or so nearly so, as to endanger the vitality of the tooth from the pressure caused by introducing the gold. Two points are indispensable in stopping the sides of front teeth—space to enable the dentist to operate, and depth of cavity to retain the stopping. If that portion of the tooth is filed away, which if suffered to remain, would have given sufficient depth, the latter requisite must be obtained by excavating; and thousands of beautiful front teeth have been entirely ruined by these injudicious excavations, which might have been permanently saved if wedging had been at first employed to gain the space required, leaving the enamel entire as the surrounding wall of the plug.

I have rarely failed to obtain a smooth wall by taking off the thin edge of the enamel to such an extent that an even surface could be gained. It is now more than seventeen years since I first performed the operation, and I do not remember a single instance where the death of the tooth has been occasioned by wedging. I have seen the vitality of teeth sometimes destroyed that had been wedged, but a careful examination could always trace it to the pressure of the gold upon the nearly or altogether exposed nerve. I do not claim any great merit for the discovery of the use of wedges; but I consider the process important and valuable alike to patient and the careful dentist.

About the year 1835, a young patient of mine was in Paris, whose father wrote to me that her two front teeth were beginning to overlap each other, and desired to know what I would recommend in such a case. I wrote to him not to have the teeth filed, but to apply wedges, and requested him to call upon Mr. Brewster. Mr. B. performed the requisite operation most satisfactorily without filing, and sometime afterwards I received a very excellent letter from him on the subject, making reference to this case and alluding to experiments which he had made with India rubber, which he advised me to try in similar cases.

Objections to this mode of operating that it induces irritation and inflammation have originated, I think, from means which have been injudiciously and improperly had recourse to. I knew a gentleman who is exceedingly nice and circumspect in his attention to his teeth. He has been for fifteen years in the habit of opening them by introducing folds of linen for the purpose of having them examined on their lateral surfaces without the slightest inconvenience or pain. I have worn wedges between my molars two and three months at a time.

We have many cases in which wedges are not required when the teeth are so much decayed as to make it necessary to cut away with the file or other instrument a considerable portion of the tooth. In doing this, sufficient room is commonly gained for the purposes required; but in such instances, I file no more than is absolutely necessary to procure a good surface, and if sufficient space is not thus obtained, a single day or so with a wedge will suffice to get all the width that may be needed.

*(To be Continued in next Numher.)*

